# Cancer imaging in the era of precision treatment: present and future

**DOI:** 10.1186/1470-7330-14-S1-O30

**Published:** 2014-10-09

**Authors:** Dow-Mu Koh

**Affiliations:** 1 Royal Marsden Hospital, Sutton, UK

## 

The landscape of cancer imaging is being shaped by advances in oncological treatment. As the genetic and molecular aberration of cancers and cancer subtypes are being elucidated, specific cellular pathways and associated molecular receptors are recognized as potential pharmacological targets to modify or arrest cancer growth. In the past two decades, such targeted therapies are being developed and are progressively being introduced into the clinics. Broadly, they include monoclonal antibodies (-abs) targeted against circulating molecules or membrane receptors; and small molecules (-ibs) which act intracellularly on biochemical pathways.

A number of molecular targets have been identified in the past decade in a range of tumours (including prostate, gastrointestinal stromal, colorectal, melanoma, chronic myeloid leukaemia, renal, lymphoma and myeloma) for which there are now approved and effective drugs directed at specific molecular receptors or cellular pathways. Many of these act by inhibiting signal transduction, blocking the signal between receptor activation and cellular proliferation; while others modify signalling proteins that regulate cellular functions. Another common mode of action is inhibiting neovascularization (antiangiogenic or antivascular), which in turn arrests tumour growth. There are also tumour vaccines and immunotherapies which stimulate cytotoxic response to cancer cells.

Although target therapies have been employed with varying success in cancers, the complexity of tumour escape pathways and the recognition that monotherapy alone may not be effective, means that it has not been possible to fully individualise treatment for patients. This remains the ultimate goal of cancer management. However, accurate genetic and molecular tumour profiling is already helping to identify tumour subgroups enabling more precise treatment to be prescribed.

Precision treatment aims to give the right drug (or combination of drugs) to the right patient at the right time to optimise tumour cell kill and minimise drug toxicity. In a wider context, precision treatment is also evolving in surgery and radiotherapy. The advent of robotic surgery allows more precise control of surgical dissection in some anatomical areas. In radiotherapy, the evolution from simple external beam treatment to state-of-the-art stereotactic body radiotherapy and cyberknife treatment means that high-dose or hypo-fractionated radiation can be delivered precisely to small target volumes thereby maximizing cell kill and reducing peri-tumoural complications.

Cancer imaging remains at the heart of this approach, as precision treatment mandates precision imaging. In the era of targeted therapy and precision treatment, cancer imaging aims to accurately depict disease burden, provide the roadmap for treatment planning, enable novel assessment of treatment response and yield prognostic information. Although conventional imaging remains important; molecular, functional and hybrid/multiplex imaging are being developed and utilised.

The depiction of disease sites and metastatic burden has implications for disease staging and prognostication. Body diffusion-weighted MRI (DWI) is now widely used as a contrast mechanism to improve disease detection at initial diagnosis and at disease relapse; especially for liver, peritoneal and bone metastases. State-of-the-art high spatial resolution CT and MRI provide detailed anatomical roadmaps for surgical and radiotherapy planning. The deployment of functional and molecular imaging yield additional biological information that can be used to define biological target volumes for precise radiotherapy planning. The portfolio of current imaging techniques also enables us to observe treatment induced changes or complications; and advance our knowledge in the effects of radiation, cytotoxic and targeted therapies on normal tissues.

As many targeted treatments may be effective without significant tumour shrinkage, conventional size-based measurement critieria are often inadequate for determining treatment effectiveness. Not surprisingly, quantitative CT, MRI and radiotracer techniques are being used to provide objective measurements of tumour properties such as tumour perfusion, water diffusion, metabolism, hypoxia, cellular proliferation and cell death. As these techniques become standardised and more widely adopted, they can provide both unique and corroborative information about tumour behaviour.

Nonetheless, there are significant challenges and opportunities ahead. First, better understanding of heterogeneity within and between tumours remains an unmet need. Repeated lines of treatment result in differential tumour clonal evolution, leading to sub-fractions of tumour cells which become refractory to treatment. A better understand of the onset and nature of resistant phenotypes is likely to contribute to the design of more effective future treatment regimes. Second, there is a huge opportunity to develop multiplex imaging on hybrid imaging systems (e.g. PET-MRI) to improve our understanding of intra- and inter-tumour variations, and the identification of imaging phenotypes associated with treatment resistance and poor prognosis. Such information can be combined with genomics and other molecular profiles on a common informatics platform to provide a rich matrix for data mining and hypothesis testing. Third, more translational research using human analogous animal models, where available, would make a significant contribution to the understanding of the disease and treatment by providing histological and molecular validation for both mechanistic and therapeutic drug studies. Fourth, it is necessary to align future workforce and imaging practices between the historical domains of radiology and nuclear medicine. The development of an integrated research and service strategy between these disciplines would be critical for the future success of multimodal multiplex imaging. Last but not least, in an age where imaging sciences are being increasingly commoditized, it is important for imaging specialists to add value by active engagement in clinical decision-making and management discussions, so that our practice remains current and relevant to the rapidly changing oncological practice.

